# Lip reading role in the hearing aid fitting process

**DOI:** 10.1016/S1808-8694(15)31129-0

**Published:** 2015-10-20

**Authors:** Ana Helena Bannwart Dell'Aringa, Elisabeth Satico Adachi, Alfredo Rafael Dell'Aringa

**Affiliations:** 1 Specialization, Speech Therapist at the Speech Therapy Sector in Otorhinolaryngology at Marilia Medical School; 2 Specialization, Speech Therapist at the Speech Therapy Sector in Otorhinolaryngology at Marilia Medical School; 3 Doctor - Professor, Chief of Otorhinolaryngology at Marilia Medical School

**Keywords:** hearing aid, hearing loss, lip reading

## Abstract

**Summary:**

Lip reading (LR) is unconsciously practiced as we communicate and has currently been widely used in the assessment of hearing impaired people. The hearing challenged individual is able “to read” lip position and thus interpret the speech sounds of the speaker; however, it is very likely that the best lip reader can only catch 50% of the words uttered.

**Methodology:**

30 individuals of both gender, with age ranging from 27 to 89 years, carriers of moderate bilateral sensorineural hearing loss. The assessment encompassed speech recognition test of monosyllable words in four situations: without hearing aid (HA) and LR; without HA and with LR; with HA and without LR; and with HA and LR.

**Results:**

we noticed an improvement in the percentage of correct answers in 93.5% of the patients with HA and LR when compared to those patients in the other situations.

**Conclusion:**

Lip reading is an important communication strategy for those with hearing impairment, and it can support the hearing aid fitting process.

## INTRODUCTION

Communication difficulty is considered the most important consequence of hearing impairment. Hearing impaired individuals try to minimize this difficulty by using some mechanisms in order to have a better understanding of what is being said. Thus, they send the message back to the person they are talking to, in a much easier way. These mechanisms are called by Speri (2000) “communication strategies”^1^.

According to Boéchat (1992), communication strategies are a set of given attitudes that work as facilitating agents for the message to be easily received, both in a visual and hearing way^2^.

The same author organized communication strategies in groups, according to their nature and she classified them into cognitive, interventional, mechanic, palliative, remedial, waiving and simulative ones.

Among the cognitive strategies, which aim to rescue the content of the message, there is lip reading (LR).

Besides using communication strategies as facilitating agents for communication effectiveness, the use of Lip Reading becomes essential for this purpose. According to Kozlowski (1997), the visual processing of speech is used even among listeners, as part of speech perception^3^. This process takes place mainly when the signal/noise relationship is unfavorable, since the phonemes are hidden by the noise, only being audible by the listener.

For Demorest, Bernstein (1992) lip reading is the most prevalent expression within the cognitive type of strategy, where individuals use several clues to understand speech, as for example, paying attention to facial expressions, recognition of gesture clues, paying attention to environmental clues and others^4^

The use of lip reading is unconsciously done when we communicate watching for facial expression, gestures, change of posture and clues that show us ways to decode the information, and it is being currently used when assessing hearing impaired individuals.^5^

The hearing challenged individual is able “to read” lip positions and thus interpret the speech sounds of the speaker; however, it is very likely that the best lip reader can only catch 50% of the words uttered, since many phonemes have an invisible articulation and others have the same articulation.^6^

For Russo (1999), it is necessary to select, indicate and adapt hearing aids together with global audiologic rehabilitation programs, in order to minimize the psychosocial reactions of older people to the aspects mentioned above, helping this hearing impaired person and his/her relatives^7^.

Therefore, this study aimed to research lip reading benefits during the hearing aid fitting process in adults.

## MATERIAL AND METHOD

This study was done at the Speech Therapy Sector at the Otorhinolaryngology Department at Marilia Medical School (FAMEMA), and approved by the Research Ethics Committee under protocol # 322/04. Thirty one patients were selected with ages ranging between 27 and 89 years (average= 65.6 median = 71), whose threshold tonal audiometry indicated symmetric bilateral hearing impairment, both sensorineural and moderate, being these two the inclusion factors. All the patients were being submitted to hearing aid selection and indication process for the first time, and they have never had a hearing aid test previously, exclusion factors.

Data collection was done through the analysis of medical records. We selected medical records from patients meeting the inclusion and exclusion factors described above.

We collected data relative to a speech perception test of monosyllable words (Lacerda et al., 1976) in 4 situations, which were presented as follows^8^:

- Without HA and without LR (1st situation);
- Without HA with LR (2nd situation);
- With HA without LR (3rd situation);
- With HA with LR (4th situation);

This specific procedure is used as routine for hearing aid selection on this institution.

The different tests were applied orally by the same investigator, with an intensity of around 70/75 dB, in a well-lit room and with minimum noise. The distance between the subject and the investigator was 1 meter.

Davis, Silvermann's classification (1970) was used to determine the degree of hearing impairment^9^.

## RESULTS

Results were analyzed in a descriptive manner, according to statistical orientation.

On Table 1, it is possible to observe the correct answer percentage obtained by each patient, in all 4 situations of the speech perception test.

**Table 1:** Depicts the percentage of right answers per patient in the speech perception test in each assessment situation. (n=31)

Patient	TPF without AASI And without LOF	TPF without AASI And with LOF	TPF with AASI And without LOF	TPF with AASI And with LOF
1	0%	20%	32%	52%
2	16%	24%	52%	80%
3	64%	76%	76%	88%
4	0%	21%	44%	64%
5	20%	36%	60%	80%
6	12%	24%	68%	72%
7	16%	24%	60%	68%
8	0%	28%	40%	76%
9	16%	100%	88%	100%
10	4%	16%	36%	64%
11	24%	52%	76%	88%
12	24%	68%	52%	84%
13	12%	24%	60%	72%
14	8%	56%	20%	64%
15	28%	72%	76%	88%
16	0%	32%	68%	80%
17	0%	4%	60%	68%
18	16%	40%	48%	72%
19	16%	24%	60%	68%
20	16%	56%	52%	80%
21	12%	68%	68%	72%
22	24%	40%	60%	76%
23	0%	16%	32%	80%
24	20%	60%	44%	60%
25	8%	12%	48%	60%
26	0%	20%	56%	68%
27	12%	12%	52%	68%
28	4%	24%	16%	52%
29	32%	68%	80%	86%
30	0%	24%	80%	88%
31	8%	28%	56%	76%

Situation number 1 presented a higher degree of difficulty for the hearing impaired individual, since the patient was without HA and without LR.

When comparing situations 1 and 2, without HA, without and with LR, it can be noticed that 100% of the patients (31 patients) scored higher using LR. The same happened when we compare the two following situations, 3 and 4, with HA, with and without LR, since 100% scored better using HA.

Results such as these, are also similar between situations 1 and 3, without LR, with and without HA, it was confirmed that 100% of patients scored better using HA.

However, in situations 2 and 4, where patients used lip reading both with and without using HA, 2 patients obtained the same score in both situations.

And comparing the last situation with the rest, 93.5% improved using HA and LR.

## DISCUSSION

We will now establish comparison relationships between the different situations.

When we compare the first two situations, where almost all individuals did not have HA, 97% of them obtained an improved rate on speech recognition when they did LR.

Therefore, we can observe that using LR only, most hearing impaired patients can benefit from this communication strategy.

Now, regarding the two following situations, 3 and 4, where all individual used HA, on situation 3 without LR and on situation 4 with LR, 100% of patients assessed obtained a better percentage on getting the words right when doing LR.

This type of data is similar to those reported by Schartz et al. (2004), who stated that watching a speaker's lips helps the listener to hear better, and as a consequence, to understand better^10^.

Between situations 1 and 3, where they did not have the help of LR, we can notice a better score on 100% of individuals assessed, when they used HA.

Silva et al. (2002) described that the use of hearing aids, is important to improve the cognitive functions in older hearing impaired patients^11^.

The same thing did not happen between situations 2 and 4, where almost every individual did LR, situation 2 was without HA and situation 4 with HA, since 6.5% obtained the same result, both with and without HA.

Situations 2 and 3 called our attention, since between them 16% of individuals scored higher on the second one, where they did not have HA but did LR.

It is likely that this fact happened because the test with HA was done with hearing impaired patients who had never used or tested HA previously, but knew how to do LR as a communication strategy, being the hearing stimulus on a second level.

This could also be justified by the conclusion reached by the study done by Boéchat (1992), where it is said that those individuals dealing better with hearing impairment, used the communication strategies more often 2.

Blamey et al. (1989) described that when hearing does not offer proper sensorial information on speech, the visual and tactile aspects can be used as additional or alternative sensorial channels, and therefore, they should be used with the aim of increasing the communication potential of those with hearing loss ^12^.

Hull (1992) talked about the importance of introducing older patients to hearing difficulties on a hearing rehabilitation program, where LR training would be emphasized, making up for the communicative difficulties found when they only use HA ^13^.

In a similar study, authors concluded that HA and LR together, offer a significant improvement in recognizing consonants. Whereas LR offers information regarding the point of articulation, HA helps with its point and manner, and with information on vowels^14^.

Other studies such as this one, were not found on compulsory literature, however, on a paper done by Mello et al. (2004), the authors reported that 100% of assessed individuals used the cognitive strategies, LR among them, both with HA and without HA^15^. And they concluded that this was the most used fact, due to its spontaneity and its easy application, since it is widely used, even among normal listeners.^16^

Marques et al. (2004) showed on their study, that after a hearing rehabilitation program, aiming to assess the capability to integrate visual and hearing clues, through phoneme training, only one subject did not improve the speech perception of monosyllable words.

With this study, we can state the importance of LR as a mechanism to facilitate speech understanding and keeping a successful conversation. From a general point of view, LR brings benefits to patient's well-being with more self-esteem and, therefore, a better social life.

An important factor we should highlight is the fact that this study was carried out in a quiet and lit setting, which makes easier the use of these strategies. When the patient is in a noisy place where noise is louder than speech, the HA system may not be effective and the strategies play a significant role for communication.

## CONCLUSION

From the results obtained with this study one can conclude that:

Most individuals obtained a better score in the speech perception test when they used lip reading, both with and without HA.

The most beneficial situation for hearing impaired individuals was situation number 4, where they had HA and LR.

Lip reading is an important communication strategy for hearing impaired individuals and its recommendation helps the HA fitting process.
